# Physical activity is associated with cardiac autonomic function in adolescent men

**DOI:** 10.1371/journal.pone.0222121

**Published:** 2019-09-06

**Authors:** Jaakko Tornberg, Tiina M. Ikäheimo, Antti Kiviniemi, Riitta Pyky, Arto Hautala, Matti Mäntysaari, Timo Jämsä, Raija Korpelainen

**Affiliations:** 1 Department of Sports and Exercise Medicine, Oulu Deaconess Institute Foundation, Oulu, Finland; 2 Center for Life Course Health Research, Faculty of Medicine, University of Oulu, Oulu, Finland; 3 Medical Research Center Oulu, Oulu University Hospital and University of Oulu, Oulu, Finland; 4 Center for Environmental and Respiratory Health Research, University of Oulu, Oulu, Finland; 5 Research Unit of Internal Medicine, University of Oulu, Oulu, Finland; 6 Research Unit of Medical Imaging, Physics and Technology, Faculty of Medicine, University of Oulu, Oulu, Finland; 7 Cardiovascular Research Group, Division of Cardiology, Oulu University Hospital, University of Oulu, Finland; 8 Centre for Military Medicine, the Finnish Defence Forces, Helsinki, Finland; 9 Diagnostic Radiology, Oulu University Hospital, Oulu, Finland; University of Brasilia, BRAZIL

## Abstract

**Introduction:**

Moderate to vigorous physical activity (MVPA) has been shown to be associated with autonomic regulation of the heart measured with heart rate variability (HRV). Only a limited amount of studies have examined this relationship among adolescents, and the effects of increasing PA on HRV is not well established. The aim of this study was to investigate how overall self-reported PA associates with HRV in a large population of adolescent men.

**Methods:**

The study was part of the Finnish MOPO study consisting of 3629 young men (mean age 18, SD 1 years) enrolled for military call-ups in 2009–2013. Overall PA, including both the intensity and frequency of habitual exercise, was assessed by a questionnaire and the respondents categorized into four groups of PA (low, moderate, high and top). Short-term HRV, physical performance and body composition were measured.

**Results:**

HRV, as indicated by mean ln rMSSD, increased according the PA categories as follows: low (3.65 ms (SD 0.7), p<0.001 vs. other groups), moderate (3.78 ms (0.6) p<0.001), high (3.85 ms (0.6) p<0.001) and top activity (3.93 ms (0.6) p<0.001) According to the multivariable linear regression analysis, a significant positive relationship (β = 0.129, p<0.05) was observed between self-reported PA and ln rMSSD independent of body mass index, waist circumference and fat percentage.

**Conclusions:**

Physical activity was positively associated with cardiac autonomic regulation, in adolescent men. A linear increase in HRV according to PA was observed, suggesting that even slight increments in PA might be beneficial for cardiac autonomic regulation The results emphasize the importance of physical activity in improving cardiac health in young people.

## Introduction

Despite the fact that physical activity (PA) is positively associated with many health benefits and decreased risk of morbidity and all-cause mortality [1, 2], most people of various ages do not meet the recommendations for daily PA [3, 4] The World Health Organization [5] recommends that young people under the age of 18 should engage in at least 60 minutes of moderate-to-vigorous daily PA (MVPA). However, in Finland, only 9% of 16-to-19-year-old people met this recommendation [6].

The autonomic nervous system (ANS) plays a central role in regulating human bodily functions. Heart rate variability (HRV) is a non-invasive tool for assessing beat-to-beat changes in HR dynamics. HRV reflects the output of sympathetic and parasympathetic (vagal) activity of ANS, and the vagal HRV influence dominates under resting conditions in healthy persons [7]. Lower levels of vagal HRV indicate ANS imbalance and have been associated with various cardiovascular and metabolic diseases, including type 2 diabetes, metabolic syndrome, hypertension and coronary heart disease [8–10], as well as all-cause mortality [11, 12].

Increasing PA [13] and regular training [14] among adults is associated with increased parasympathetic activity and higher overall HRV suggesting improved cardiac autonomic regulation. The amount and intensity of PA is influential, and some of these studies suggest that moderate to vigorous PA (MVPA) is required for observing an increase in vagal indices of HRV [15, 16]. There are only a limited amount of studies conducted among adolescents [16–20] or young adults [21] that have examined the association between PA and HRV. Most of the previous studies have involved less than a hundred participants and assessed PA either by self-reports [17, 20] or objective measurements [19, 21]. Often, the categorization of the amount, or intensity of PA, has either been binary [22] or has not been distinguished, or described [19, 20]. A recent systematic review of children and adolescents (age 5–18 years old) found that despite the heterogeneity of studies, MVPA is positively associated with HRV (RMSSD) and the relationship between other levels of PA and HRV is less clear. [16] A cross-sectional study among young adults suggested that vagal modulation is enhanced only with high levels of PA [21]. Otherwise, the effects of increasing PA on HRV among adolescents, is not well established. For addressing this gap in the knowledge, assessment of self-reported PA provides a feasible tool in population studies [23]. This also enables to examine overall PA utilizing composite questions including elements of both frequency and intensity of PA.

The purpose of the study was to investigate the relationship between physical activity and autonomic cardiac regulation, measured by HRV, in a large population-based sample of healthy adolescent men at the verge of adulthood. Our hypothesis was that PA is positively related to HRV. We further hypothesized that increasing overall self-reported PA would improve HRV.

## Methods

### Participants and study protocol

The study was part of the population-based Finnish MOPO research project [24] and entailed cross-sectional questionnaires and measured data collected at the annual call-ups in the city of Oulu each autumn, from 2009 to 2013. The call-ups were mandatory for all men in Finland in the year when they turned 18 years. Thus, they provided a large, population-based representative sample of young men. In total, 5,824 young men participated in the call-ups and were invited to participate in the study. Ultimately, the study population consisted of 3,629 adolescent males whose HRV had been recorded and who had completed a questionnaire on their health, lifestyle and PA. Due to poor HRV signal quality, 234 men were excluded. Thus, the total participation rate of the study was 58% (n = 3,395), and it ranged from 54% to 68% annually.

The study was conducted according to the Declaration of Helsinki and the Ethical Committee of Northern Ostrobothnia Hospital District (ETTM123/2009) approved it. The participants had the right to refuse to participate in it or to withdraw from it at any time without any effects on their future health care or military service. Written informed consent was obtained from all individual participants included in the study.

### Measurements

#### HRV measurements

All HRV measurements were performed between 11:00 am and 3:00 pm and were conducted in tranquil conditions to minimize any stress. During the measurement, the participants were instructed to lie down and relax for five minutes before the recording started. A heart rate monitor (Polar RS800; Polar Electro Oy, Kempele, Finland) was used to record RR intervals, and the timing resolution was 1 ms. The duration of each measurement was 5 minutes, and the participants were allowed to breathe spontaneously during the recording.

The HRV data were analyzed using Kubios HRV software [25]. Prior to the analysis, the data were edited by deleting artefacts and ectopic beats, and the last 4 minutes of recording time were used for analyses. The HRV was evaluated using several time-domain and frequency-domain analyses according to the guidelines of the Task Force of the European Society of Cardiology and the North American Society of Pacing and Electrophysiology [7]. The computed time-domain parameters included the mean heart rate (HR), the mean RRi (RR interval), the standard deviation of the RRi (SDNN) and the root mean square of successive normal RRi differences (rMSSD). The frequency-domain parameters were peak low-frequency (LF, 0.04–0.15 Hz) and high-frequency (HF, 0.15–0.4 Hz) bands and the ratio of LF to HF powers (LF/HF).

*Self-reported physical activity*. The participants completed a health and lifestyle questionnaire that included items on health, health behavior, amount of PA and self-rated physical fitness. They were asked to select the best option for the self-estimated PA level during the past six months: “low” (described as no regular PA, occasional walking, < 0.5 h/week), “moderate” (regular recreational PA or moderate occupational PA, 0.5–2 h/week), “high” (regular heavy physical exercise, 2–4 h/week), or “top” (heavy physical exercise at least 5 times a week, > 4 h/week) [26].

#### Physical performance measurements

Height and waist circumference were measured using a ruler, and body composition (body mass index, fat and muscle percentage, weight) was measured using bioelectrical impedance equipment (InBody 720, Biospace Co., Ltd., 2005). Aerobic fitness was assessed using the Polar Fitness Test^™^ (Polar Electro, Finland), which predicted aerobic fitness based on resting HR, HRV, gender, age, height, body weight and self-assessment of the level of long-term PA [26]. The fitness measurement was conducted simultaneous with the HRV recording, with the study participant in the supine position following five minutes of rest. Bilateral maximal isometric grip strength was measured with a dynamometer (SAEHAN Corporation, Korea) (Bohannon, 2012). The subject was standing legs apart and elbow at a 90° angle and was advised to grip the instrument with maximum strength. The best result of two attempts per hand was recorded. The mean value of both hands was used.

#### Statistical analyses

Statistical analyses were performed using SPSS/PASW version 21 (IBM Corp., Armonk, New York, USA). Descriptive data were presented as means and standard deviations in the case of continuous variables and as numbers with percentages in the case of categorical variables. Data normality was tested using the Kolmogorov-Smirnov test, and, because of the skewed distribution of some variables, the HRV indices (rMSSD, LF power, HF power and LF/HF ratio) were transformed using natural logarithms (ln) to allow for comparison between the categories of PA.

A one-way ANOVA analysis with the Bonferroni correction was used to compare the means between different PA categories. A linear regression analysis was performed to determine the association between PA, body composition and HRV indices. Based on the detected significant univariate associations, the models were adjusted for diseases (diabetes, mental disorders, thyroid disorders, musculoskeletal diseases and asthma), and, smoking and snuffing, coffee drinking and perceived stress immediately prior to the HRV measurement (15 min or less). The level of statistical significance was set at p < 0.05.

## Results

The characteristics of the study participants across the different PA categories are presented in Table 1. Those who reported a low level of PA had higher body fat percentages and waist circumferences, but lower fitness and body lean mass percentages compared with the more physically active persons. They also reported more frequent smoking and use of alcohol and a higher prevalence of mental disorders than the categories of higher physical activity. Interestingly, the most physically active men also snuffed more often than the members of the other groups.

**Table 1:** The characteristics of the study participants (n = 3,395) according to reported physical activity level. Values are presented as means (SD) unless otherwise stated.

	Low n = 819	Moderate n = 1,229	High n = 982	Top n = 380	p value
Height, cm	178 (6)	178 (6)	178 (6)	179 (6)	0.008
Weight, kg	73.1 (16.7)	72.6 (14.5)	72.2 (11.4)	74.3 (10.4)	0.086
Body mass index, kg/m2	23.1 (5.0)	23.0 (4.3)	22.7 (3.3)	23.3 (2.8)	0.038
Body fat, %	18.8 (9.3)	17.0 (8.1)*	14.2 (6.4)*†	13.2 (5.4)*†‡	<0.001
Lean mass, %	45.5 (5.3)	46.7 (4.6)*	48.5 (3.8)*†	49.3 (3.1)*†‡	<0.001
Waist circumference, cm	83.0 (12.4)	81.5 (10.5)*	80.3 (7.9)*†	80.8 (6.2)*	<0.001
Aerobic fitness, ml/kg/min	46.9 (3.3)	50.6 (3.0)*	57.9 (5.0)*†	65.8 (6.9)*†‡	<0.001
Grip force, kg	46.8 (8.7)	48.5 (8.4)*	50.0 (8.3)*†	50.8 (8.5)*†	<0.001
Daily smoking, n (%)	224 (27.0)	237 (19.3)*	138 (14.1)*†	16 (4.2)*†‡	<0.001
Daily snuffing, n (%)	60 (7.3)	125 (10.2)	158 (16.1)*†	82 (21.6)*†‡	<0.001
Weekly alcohol consumption, n (%)	146 (17.8)	155 (12.6)*	132 (13.4)*	28 (7.4)*	<0.001
Diabetes, n (%)	5 (0.6)	7 (0.6)	3 (0.3)	2 (0.5)	0.783
Mental disorders, n (%)	122 (14.9)	83 (6.8)*	56 (5.7)*	11 (2.9)*	<0.001
Thyroid disorders, n (%)	3 (0.4)	2 (0.2)	1 (0.1)	1 (0.3)	0.633
Asthma, n (%)	62 (7.6)	84 (6.8)	59 (6.0)	36 (9.5)	0.142
Musculoskeletal diseases, n (%)	117 (14.3)	136 (11.1)	122 (12.4)	57 (15.0)	0.082

Statistical differences (p < 0.05) between groups:

* vs. low.

† vs. mod.

‡ vs. high.

Low = no regular PA, occasional walking, < 0.5 h/week)

Moderate = regular recreational PA or moderate occupational PA, 0.5–2 h/week

High = regular heavy physical exercise, 2–4 h/week

Top = heavy physical exercise at least 5 times a week, > 4 h/week

**Fig 1:**
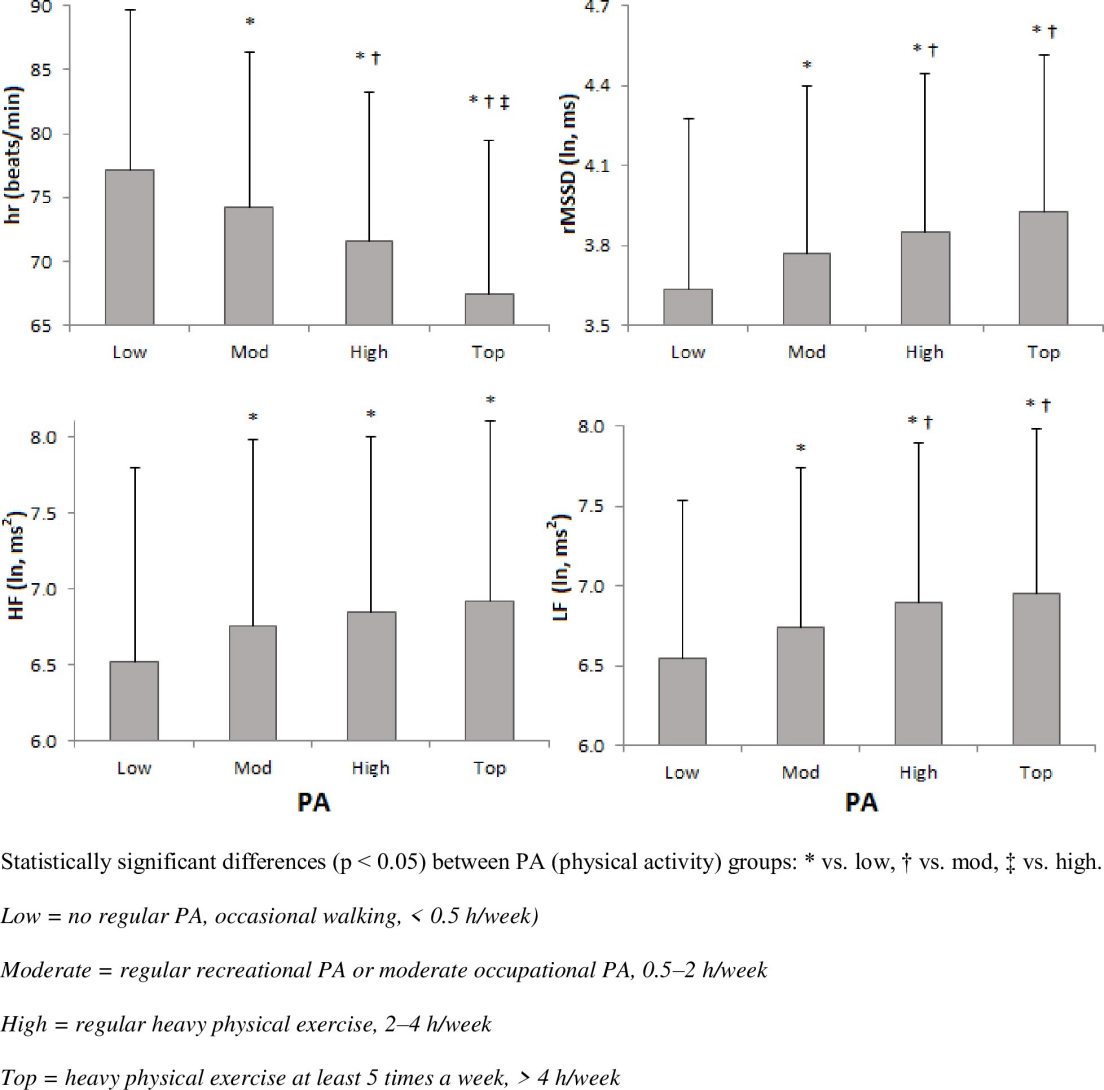
The mean (SD) HRV values according to physical activity level in young men (n = 3395).

The mean (SD) HRV values for the different PA groups are presented in Fig 1 and Table 2. Ln rMSSD increased with the PA level, and the mean heart rate reduced systematically when the PA level increased. Of the spectral indices of HRV and ln LF power were significantly higher among the two groups that reported the highest levels of PA. Among the young men with the lowest level of PA, ln HF power was significantly lower than that of the other groups. There was a significant positive relationship between PA level and SDNN. LF/HF was not significantly associated with PA.

**Table 2:** The heart rate parameters (n = 3,395) according to reported physical activity level. Values are presented as means (SD) unless otherwise stated.

	Low n = 819	Moderate n = 1,229	High n = 982	Top n = 380	p value
RRi (ms)	801 (128)	834 (128)*	864 (140)*†	922 (160)*†‡	<0.001
rMSSD (ln, ms)	3.65 (0.7)	3.78 (0.6)*	3.85 (0.6)*†	3.93 (0.6)*†	<0.001
SDNN (ms)	47.0 (22.6)	52.2 (24.4)*	54.8 (24.6)*†	57.0 (24.9)*†	<0.001
pNN50 (%)	23.6 (20.0)	27.5 (21.4)*	29.7 (21.0)*	32.9 (22.0)*†	<0.001
LFpower (ln)	6.56 (1.0)	6.74 (1.0)*	6.90 (1.0)*†	6.96 (1.0)*†	<0.001
HFpower (ln)	6.56 (1.3)	6.77 (1.2)*	6.85 (1.2)*	6.93 (1.2)*	<0.001
LF/HF (ms^2^)	1.56 (2.2)	1.56 (1.8)	1.60 (1.8)	1.70 (2.4)	

Statistical differences (p < 0.05) between groups:

* vs. low.

† vs. mod.

‡ vs. high.

Low = no regular PA. occasional walking. < 0.5 h/week)

Moderate = regular recreational PA or moderate occupational PA. 0.5–2 h/week

High = regular heavy physical exercise. 2–4 h/week

Top = heavy physical exercise at least 5 times a week. > 4 h/week

Table 3 shows the factors that were significantly associated with the HRV indices according to the multivariable linear regression analyses. A significant positive relationship between self-reported PA level and all HRV indices was revealed. The final models explained 9.1% of the variance in the mean heart rate, 5.0% of the variance in ln rMSSD, 3.6% of the variance in ln HF and 3.0% of the variance in ln LF. Waist circumference was significantly and inversely associated with ln rMSSD and ln HF.

**Table 3:** Significant predictors of HRV indices and HR in multivariable linear regression analysis concerning adolescent men (n = 3,395).

Variable	Regression Coefficient (95% CI)	SE	p value
*Heart rate*: *model R*^*2*^ *= 0*.*091*, *standard error of the estimate = 11*.*79*, *P<0*.*			
Self-reported physical activity	- 2.897 (-3.429 to—2.366)	0.271	P<0.001
Constant	84.406 (80.536 to 88.277)	1.974	P<0.001
*ln rMSSD*: *model R*^*2*^ *= 0*.*050*, *standard error of the estimate = 0*.*61*, *P = 0*.048*			
Self-reported physical activity	0.091 (0.071 to 0.115)	0.014	P<0.001
Waist circumference	- 0.005 (- 0.008 to—0.003)	0.001	P<0.001
Grip force	- 0.003 (- 0.006 to 0.000)	0.002	P = 0.049
Constant	3.970 (3.653 to 4.287)	0.128	P<0.001
*Ln HF*: *model R*^*2*^ *= 0*.*036*, *standard error of the estimate = 1*.*20*, *P<0*.001*			
Self-reported physical activity	0.112 (0.058 to 0.166)	0.028	P<0.001
Waist circumference	- 0.013 (- 0.018 to—0.008)	0.003	P<0.001
Constant	7.049 (6.462 to 7.636)	0.299	P<0.001
*Ln LF*: *model R*^*2*^ *= 0*.*030*, *standard error of the estimate = 1*.*00*, *P<0*.*			
Self-reported physical activity	0.140 (0.095 to 0.185)	0.023	P<0.001
Constant	5.857 (5.530 to 6.184)	0.065	P<0.001

* Models were adjusted for other significant variables in univariate analysis (*stress*, *smoking and snuffing (15min before)*, *coffee (15min before))*

## Discussion

The present study is the first comprehensive population-based study to provide evidence regarding the association between overall physical activity and cardiac autonomic regulation in adolescent men. The main finding was that there was a linear association between overall physical activity and the vagal autonomic regulation of the heart measured as HRV. These findings underscore the importance of PA for cardiac health already at a young age.

In our study of adolescent men, there was a positive association between self-reported PA and HRV suggesting improved cardiac regulation. This finding is in accordance with a systematic review in children and adolescent [16] and a few previous limited sized population studies among adolescents [17, 19, 20] and involving either self-reported [17, 20] or measured PA [19]. In addition, a similar association between PA and HRV has also been demonstrated for young adults [21], as well as older men and women [15, 27]. Although the previously mentioned studies suggest favorable effects of exercise on HRV, not all have detected such an association [28, 29]. These inconsistent findings probably reflect differences in study designs rendering their comparisons difficult. However, overall it is suggested that a higher amount of PA improves cardiac regulation.

Our study demonstrated a linear improvement in HRV with increasing levels of self-reported PA. Here, PA was defined as a combination of both the intensity and frequency of the performed exercise [26]. Alongside, with higher reported PA we observed a consistent increase in cardiorespiratory physical fitness which is strongly related to vagal HRV indices [30, 31]. A recent systematic review of the relationship between PA and HRV in children and adolescents (age 5–18 years old) found that despite the heterogeneity of studies, MVPA is positively associated with HRV (RMSSD) but the relationship between other levels of PA and HRV is less clear (16). Previous results involving adolescent boys (16.6 ±1.2 years old yrs.) showed that high self-reported physical activity (60 min of MVPA for at least five days during the past seven days) was related to greater parasympathetic cardiac modulation [20]. Furthermore, a study conducted among young adults showed that especially repeated bouts of vigorous intensity exercise stimulated vagal function in young adults [21]. Vigorous activity was also associated with higher HRV in middle-aged persons [15]. Similarly, each additional self-reported hour of heavy exercise increased HRV in older persons [27]. Another study suggested, on the other hand, that already moderate-intensity PA would be sufficient to modify vagal HRV among middle-aged individuals [32]. We observed that already an increase from low to moderate self-reported PA increases vagal activity. In addition, being engaged in regular heavy exercise more than 2–4 h/week did not improve HRV further. It should be noted that in our study, the increase in HRV across the different PA categories reflect differences in both the frequency, duration and intensity of exercise which individual role cannot be distinguished.

Our study provides reference values for HRV measures in the time and frequency domains in a large cohort of adolescent men. The closest comparable study involving adolescent boys (12–17 yrs.) demonstrate similar baseline results for rMSSD, SDNN and LF and HF power [20]. We detected a negative association between obesity factors (waist circumference, fat percentage, BMI) and vagal activity (ln rMSSD), which is consistent with findings obtained from adolescent boys [20], young adults [29] or adults [33, 34]. However, according to the multiple linear regression analyses, the association between PA and HRV was independent of BMI, waist circumference and fat percentage. These results underline the importance of PA in cardiac autonomic regulation irrespective of body composition.

Our study included both the spectral and time domain analysis of HRV. Of the time-domain parameters especially rMSSD, reflective of parasympathetic activity [7], increased in parallel with augmented PA. The related parameters, SDNN (reflective of total variability) and pNN50 showed a similar, but non-significant trend according to PA. The results for the frequency-domain HRV indices (LnHF power, LnLF power) resembled the responses of the time-domain parameters. LnHF power, which has been shown to reflect parasympathetic modulation [7], was lower among the most inactive compared with those engaged in higher levels of PA. LF power, which includes both parasympathetic and sympathetic modulation, was positively associated with PA level. However, LF power was similar between the high and top categories of PA. LF/HF ratio was not significantly related to PA.

Assessment of HRV provides a noninvasive tool for the evaluation cardiac autonomic regulation. Resting HRV is lowered in many disorders, including hypertension, dyslipidemia, obesity and diabetes, and reflects cardiovascular functioning and cardiac health [8]. Regular training [14, 35] as well as PA [13], have been shown to shift HRV towards increased vagal dominance. Our study suggest that even moderate PA is sufficient for augmenting HRV among adolescent men. This observation underpins the importance of PA for improving cardiovascular health at an age preceding the development of manifest lifestyle-related cardiovascular disorders. The information is relevant in any activities promoting physical activity in youth.

### Strengths and limitations of the study

The major strength of this study was the large, population-based sample of adolescent men of a specific age. In our study, the total participation rate was high considering that, though the call-ups were mandatory for all men, participation in the study was voluntary. An additional strength is also the strictly controlled conditions when performing the HRV assessments.

A limitation of the study was that the assessment of PA was based on self-reports which are feasible methods commonly used in epidemiological settings involving youth [23]. However, some bias may be involved in the recall of the activity retrospectively, and over a relatively lengthy period. In addition, distinguishing for the types of habitual physical activity could have brought additional insight to the interpretation of the results. Some uncontrollable behavioral factors, such as coffee and lunch offered by Finnish Deforce Forces, prior to the HRV measurement might have affected the results. However, adjustments for self-reported coffee drinking, smoking or snuffing and stress immediately prior to the HRV measurements in our analyses help control for this type of information bias.

## Conclusions

This population-based study among adolescent men showed that cardiac autonomic regulation improved according to the increase in the frequency and intensity of physical activity independent of body composition. The results emphasize the role of physical activity for cardiac health already at young age. The results can be utilized in promoting physical activity and reducing adverse health effects associated with a sedentary lifestyle among youth.
